# Neuroimaging in Adolescents: Post-Traumatic Stress Disorder and Risk for Substance Use Disorders

**DOI:** 10.3390/genes14122113

**Published:** 2023-11-23

**Authors:** Yasameen Etami, Christina Lildharrie, Peter Manza, Gene-Jack Wang, Nora D. Volkow

**Affiliations:** Laboratory of Neuroimaging, National Institute on Alcohol Abuse and Alcoholism, National Institutes of Health, Bethesda, MD 20892, USA; yasameen.etami@nih.gov (Y.E.); christina.lildharrie@nih.gov (C.L.); peter.manza@nih.gov (P.M.); nora.volkow@nih.gov (N.D.V.)

**Keywords:** adolescence, SUDs, PTSD, epigenetics, memory, neuroimaging

## Abstract

Trauma in childhood and adolescence has long-term negative consequences in brain development and behavior and increases the risk for psychiatric disorders. Among them, post-traumatic stress disorder (PTSD) during adolescence illustrates the connection between trauma and substance misuse, as adolescents may utilize substances to cope with PTSD. Drug misuse may in turn lead to neuroadaptations in learning processes that facilitate the consolidation of traumatic memories that perpetuate PTSD. This reflects, apart from common genetic and epigenetic modifications, overlapping neurocircuitry engagement triggered by stress and drug misuse that includes structural and functional changes in limbic brain regions and the salience, default-mode, and frontoparietal networks. Effective strategies to prevent PTSD are needed to limit the negative consequences associated with the later development of a substance use disorder (SUD). In this review, we will examine the link between PTSD and SUDs, along with the resulting effects on memory, focusing on the connection between the development of an SUD in individuals who struggled with PTSD in adolescence. Neuroimaging has emerged as a powerful tool to provide insight into the brain mechanisms underlying the connection of PTSD in adolescence and the development of SUDs.

## 1. Introduction

As PTSD can result in changes in the neural pathways of adolescents, it raises the question of potential connections to developing an SUD. During adolescence, physical and emotional changes occur that shape an individual’s development [1]. Different brain regions develop at various rates—including some that do not fully develop until the mid-20s—which have consequences for behavior [2]. The plasticity of the brain makes it susceptible to various internal and external influences until the mid-twenties [3]. Since adolescence is a period of high vulnerability for the emergence of mental illness, understanding brain developmental trajectories can provide explanations for a range of important behaviors including academic performance, sociability, and potential criminal justice involvement.

Trauma is also more likely to occur during childhood and adolescence than at any other time of life for individuals with SUD, and 24–30% of adolescents with PTSD have comorbid SUDs [4]. Adolescents with SUD reported a threefold higher rate of traumatic events and a fivefold higher prevalence of PTSD following traumatic events, compared to the general adolescent population [5]. SUDs can emerge as a coping mechanism for PTSD, and on their own have the potential to create complications in the physical and mental wellbeing of an individual.

Here, we review neuroimaging studies that shed light on the neural vulnerability for the development of an SUD after a diagnosis of PTSD in adolescents. We discuss possible preventive strategies to lower the occurrence of SUDs in diagnosed youth. This review also aims to raise awareness about the need for accurate diagnosis and treatment of PTSD in adolescents, which could lower the comorbidity of PTSD and SUDs and help prevent SUDs.

We begin by providing insight into the development of the adolescent brain, then move to review PTSD, its risk factors, and its effects on the limbic system and brain network connectivity patterns. We take the same approach for SUDs. Lastly, we discuss the foundational and recent studies on PTSD and SUDs in adolescents, epigenetics, and identified research gaps in the literature.

Between the months of May–August 2023, we searched Google Scholar and PubMed using the following terms: “Addiction OR PTSD OR SUD OR PTSD and SUD in Adolescents OR fmri OR Neuroimaging in Adolescents with PTSD”. We restricted our search to articles that were published in English and between the dates of 1991–2023. Additional articles were identified via recursive reference searching and previous knowledge.

## 2. Adolescence

### 2.1. Development of the Adolescent Limbic System

Adolescence is marked by increases in brain plasticity and behavioral changes, with rates of psychopathology peaking at this life stage [6,7]. Although there has been much debate about defining the phase between childhood and adulthood, the UN Convention on the Rights of the Child defines childhood as ages 0–18 years and adolescence as ages 10–19 years [8]. As the definition of adolescence and childhood overlap between ages 10–18, we refer to all study populations in the 10–18 ranges as adolescents, and populations less than 10 years old as children. Adolescents who have experienced stress are at a higher risk for developing psychopathology [9]. Gaps exist in the current understanding of adolescent brain development and clinical research that focuses on adolescents struggling with psychiatric disorders [10].

The limbic system has been heavily researched throughout the years. Since psychiatric disorders are associated with brain structural changes, it is important to understand this trajectory during adolescence [11,12,13]. For instance, typically developing adolescents show lower gray matter volume in the insula and medial/lateral orbitofrontal cortex than adolescents with social anxiety disorder [3,14,15]. Higher gray matter volume in social anxiety has been interpreted as an impairment in normal maturational processes, whereby gray matter typically decreases as a result of pruning redundant synapses [16,17]. White matter volume displays a consistent linear increase through adolescence, peaking in early adulthood [18]. Researchers also found that compared to those with social anxiety disorder, those without diagnosed psychiatric disorders had greater structural covariance (a measure of interconnectedness, and thereby structural integrity, between brain regions) in the fronto-limbic system, a network of regions essential for cognitive control [15].

Myelination rapidly increases in early childhood and progresses throughout adolescence and adulthood [19]. A recent diffusion tensor imaging study found that among typically developing adolescents, decreased fractional anisotropy (FA) and increased mean diffusivity (MD) in the cortico-limbic white matter tracts (indicative of lower levels of white matter integrity), correlate with severe internalizing and externalizing behaviors [20]. Interestingly, the effects were most notable in the cingulum and uncinate fasciculus, regions that are commonly impacted by various mental health disorders [20].

### 2.2. Adolescent Memory

Recent neuroimaging research has studied the development of brain regions involved in memory. For example, research studying adolescent mental health disorders has found impaired episodic memory and smaller hippocampal volumes, indicating that just as in adults, the hippocampus is crucial for memory processes [21]. Episodic memory shows a nonlinear development throughout adolescence [22]. Although studies have found that memory performance consistently increases until about eight years of age, reports are inconsistent for subsequent years. Riggins [23] found that memory development continues to steadily increase after eight years old, whereas Picard and colleagues [24] reported that performance stagnates after age nine. Likewise, neuroimaging studies show discrepant conclusions about the brain development underlying adolescent episodic memory. This is in part because different studies use different tasks and often focus on the development of distinct brain regions [22]. Additionally, there are external factors that influence memory such as hormonal and sex differences that occur during puberty, intellectual ability, and the social determinants of health [25,26,27], as well as different studies may differ in their sample demographics.

The amygdala has also gathered much attention, as it is a processing center for emotions, connecting them to memories and learning. Traumatic stress in adolescence can cause morphological changes in neurons in the amygdala [28]; it might also impact the expression of the epigenetic marker H3K9me2 and decreases transcription levels of the brain-derived neurotrophic factor (*Bdnf*) gene, which promotes dendrite development and synaptic growth [28]. These pathological changes can increase the risk of mental disorders [28].

More studies are also emerging that examine changes to brain network connectivity during adolescence. In a functional magnitude resonance imaging (fMRI) study examining brain connectivity during a verbal working memory task, adolescents compared to children showed increasing functional connectivity as cognitive load increased [29]. Another study found that adolescents at risk for working memory deficits had decreased connectivity between the left frontal operculum and the anterior cingulate gyrus compared to the control group [30]. A more recent dynamic functional connectivity study found that brain states with high activity in the frontal-parietal network (FPN) during working memory were short-lived and recurring [31]. This information supports previous research showing the significance of FPN organization for retaining task-related information, leading to greater cognitive effort as the working memory load progresses [32,33,34].

## 3. Post-Traumatic Stress Disorder

### 3.1. Risk Factors

Post-traumatic stress disorder (PTSD) is a condition that can develop after a stressful, traumatic, or overwhelming event that may involve actual or potential injury or death. It is a complex disorder that cannot be defined strictly as a fear response, but rather as a variety of determinants that culminate after the traumatic event that maintains the disorder. Symptoms of PTSD include experiencing the event in the form of nightmares, avoidance, emotional numbing, or a high level of arousal. There are some defining clinical features of PTSD, as it is associated with stress, however, most individuals who are placed in extremely stressful situations will not develop PTSD. PTSD is similar to various other psychiatric disorders as its onset occurs after a stressor and its manifestation is even more likely in vulnerable individuals [35]. Childhood maltreatment is one of the most common causes of PTSD in adolescents [36].

When considering risk factors for developing PTSD, there are three main categories, pre-trauma, peri-trauma, and post-trauma. Not all individuals develop PTSD after experiencing a traumatic event, making these risk factors crucial for understanding the development of psychopathology. Pre-trauma factors can include age, gender, and race/ethnicity, peri-trauma factors include duration/severity of trauma experience, and post-trauma factors can include access to resources and social support following the traumatic event [37]. There are also factors that raise the chance of experiencing a traumatizing event and factors that raise the possibility of symptom development after the traumatic event. Through a diathesis-stress model, vulnerability factors and environmental stressors are considered together. Violence exposure can maintain PTSD symptoms over time, and boys and older youth are more likely to experience violence than girls and younger children. Sexual violence is another risk factor that becomes more common in late adolescence. Racial and ethnic differences associated with social disadvantages and a higher likelihood of exposure to adverse environments may serve as risk factors. For example, African American adolescents have a greater likelihood of experiencing violence, even though they are less likely to meet the criteria for PTSD, compared to youth from other racial groups. Previous history of violence and associating with deviant peers can serve as perpetuating risk factors for PTSD [38]. Social problems are another risk factor that can be a factor for developing PTSD in various ways, such as attachment insecurity, low social support, or social conflict [39,40,41].

### 3.2. Effects on the Limbic System

**Figure 1:**
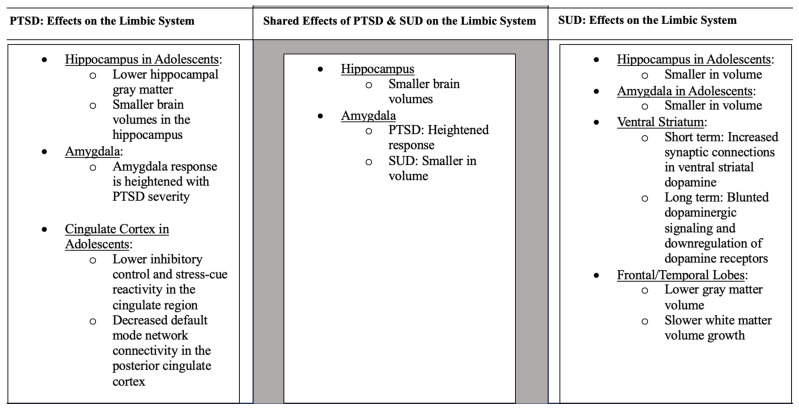
Data were pulled from Section 3.2, Section 3.3 and Section 4.2 of this review. Unless otherwise stated, the information applies to adults.

Studies have found that PTSD is associated with alterations to the fronto-limbic circuitry, specifically the hippocampus, amygdala, cingulate cortex, and prefrontal cortex [42]. For a summary of these findings, see Figure 1. Adversity faced early in life critically impacts the developing hippocampus [43]. In support, adolescents with childhood trauma show lower hippocampal gray matter compared to those without a history of trauma [44]. As previously mentioned, higher gray matter volume can indicate a malfunction in necessary synaptic pruning processes [16,17]. However, other researchers have interpreted this differently, finding that decreases in gray matter correlate with decreases in cognitive functioning, suggesting this may be a consequence of disorders triggered by environmental causes [45]. Many studies have examined memory impairments in adolescents with PTSD. Adolescents who have experienced bereavement show greater autobiographical memory impairments than those who have not [46]. Similarly, PTSD youth had lower scores on memory tests compared to healthy controls, indicating the influence of early trauma exposure at a young age on memory [47]. Children who were exposed to physical abuse and who came from households with low socioeconomic status were found to have smaller brain volumes in the hippocampus compared to those who did not have these experiences [48]. Another study found heightened activity of the hippocampus when adolescents were read trauma-related scripts [49]. With the hippocampus being a hub for memory, alterations in this essential brain region can signal long-lasting changes to neural circuitry, producing PTSD symptoms such as flashbacks.

Studies have also found that the amygdala is affected in adolescents with PTSD. One study found that compared to those without PTSD, children aged 10–16 who were diagnosed with PTSD after an earthquake were found to have higher concentrations of the following neurochemicals in the right amygdala compared to controls: N-acetylaspartate, myo-inositol, choline compounds, as well as creatine and phosphocreatine [50]. In an fMRI study, participants were shown happy, sad, neutral, and no-face primes and reported whether they produced positive or negative feelings. Results supported the idea that childhood adversity is associated with exaggerated amygdala response to negative facial stimuli [51]. However, since the traumatic experiences faced by the participants were self-reported, it could be that those who had stronger memories of traumatic events in their childhood were more likely to have a stronger amygdala response to a negative event. Additionally, it is important to note that the participants from this study were adults and were administered the Childhood Trauma Questionnaire to understand each individual’s retrospective trauma. Further, children aged 9–14 who were physically abused, faced negligence, and came from low socioeconomic households were shown to have smaller amygdala volumes than controls, similar to results for the hippocampus [48]. However, these results were from a single MRI scan, so the causal direction of effects is not yet established. Among college-aged individuals, amygdala volume in specific subregions linked to fear extinction and memory, including the centrocorticomedial complex (CMA) and the basolateral complex (BLA), were also correlated with PTSD symptomatology [52]. Additionally, listening to a description of past traumas resulted in higher amygdala activity compared to controls [49]. This finding supports previous research that the amygdala response is heightened as PTSD severity increases [53,54,55,56,57].

The cingulate cortex is essential for inhibitory control and stress responses [58]. Childhood trauma is associated with lower inhibitory control and diminished stress-cue reactivity in the cingulate region as found in a study with adolescents aged 14–17 [59]. Decreased inhibitory abilities provide a possible explanation for why traumatized youth act more impulsively than the general population. In adult participants affected by PTSD, there were significantly lower fractional anisotropy (FA) values in the cingulum than controls [60]. Alterations to this circuit in early adolescence correlate with poor cognitive and emotional functioning, potentially leading to heightened vulnerability to external stressors [61,62,63]. In another small fMRI study, participants aged 13–19 were asked to listen to a script of either a positive or negative event individualized to their past traumas. Elevated activity in the dorsal anterior cingulate cortex (ACC) was found in the individualized conditions compared to the generalized positive and negative scripts that were given as a baseline to each participant [49]. Interestingly, in a study examining adult PTSD, researchers have found gray matter decreases in the rostral ACC compared to healthy adults [60].

Other brain regions also appear to be implicated in trauma. A study exposing 74 healthy female subjects aged 18–36 to traumatic films found that more early intrusive memories correlated with lower volumes of the left insula, a common area affected in those with PTSD. Further, larger volumes of the left lingual gyrus/cerebellum and right inferior frontal gyrus/precentral gyrus correlated with greater amounts of intrusions [64]. These diverse findings point to the potentially diffuse nature of trauma pathophysiology.

### 3.3. Brain Network Connectivity

Many recent studies have also looked at the connectivity patterns in adolescents with PTSD. Supporting the hypothesis that adolescents with PTSD display similar network dysfunction as adults with PTSD, adolescents had increased connectivity within the default mode network (DMN) and decreased connectivity between the DMN and salience network (SN) and central executive network (CEN) than controls [65]. Since the DMN contributes to episodic memory and autobiographical memory, impaired DMN function may underlie some of the cognitive symptoms of PTSD. Other studies have also found disrupted nodal centrality, a measure of the significance of a node within a network, in the DMN, SN, and CEN [66]. The authors suggested that this decrease in DMN connectivity compared to controls may explain the flashbacks commonly experienced by PTSD victims, whereas the increased connectivity between the DMN and SN compared to controls may explain the exaggerated neural response during episodic memory recall in those with PTSD [65]. They also found positive and negative correlations between DMN and CEN connectivity strength, which may explain the disruptions of autobiographical memories during recollection of episodic memories.

Adolescents with PTSD also show decreased connectivity in limbic system regions [67]. Decreased DMN connectivity was found in the posterior cingulate cortex in adolescents with PTSD, a region well-studied for its functioning in visual mental imagery and autobiographical memory compared to controls [65,67]. In addition, compared to controls, adolescents aged 11–18 affected by interpersonal violence exposure and PTSD showed increases in intraparietal sulcus (IPS) cortical thickness, a crucial component of the frontoparietal cognitive control network necessary for learning and emotional processing compared to controls [68,69,70]. However, one recent study has found conflicting results. In 2020, Rinne-Albers et al., utilized MRI to investigate cortical thickness, surface area, and volume in adolescents with PTSD and a group of healthy controls [71]. Despite their initial hypothesis, there were no significant differences between the two groups on any cortical measures [71]. It is possible that since this study only examined women with PTSD after childhood sexual abuse, the conclusions may be specific to this population.

A history of adversities can predict the risk for the first onset of PTSD [72]. Different dimensions of adversity can impact brain development. Connectivity between most brain networks tends to decrease throughout adolescence, whereas youth exposed to adversity had stable connectivity over time [73]. Stability in functional brain networks could contribute to the internalization of symptoms from adolescence to adulthood [73].

Differences in the brain structural covariance network centrality of the ACC, posterior cingulate cortex (PCC), inferior frontal cortex/insula (IFC), and frontal pole (FP) are also affected in youth with PTSD [74]. With the findings of large centrality value for PCC, a key region within the episodic memory network, it could be theorized that kids with exposure to abuse continuously experience the memories of the events that caused their PTSD [67,74]. In each of these studies, the traumatic events faced by youth that result in PTSD diagnosis are associated with functional connectivity abnormalities in regions supporting memory function.

Many of these affected brain circuits in PTSD appear to be similarly impacted across a range of mental health conditions. A recent meta-analysis demonstrated that atrophy coordinates in five different psychiatric disorders aligned to a common brain network involving positive connectivity to the insula, posterior cingulate, left frontal pole, and anterior cingulate. Authors also found negative connectivity to the posterior parietal cortex, lateral occipital cortex, dorsal areas of the cerebellum, as well as the brainstem [75]. This thought introduces the idea of the shared neurobiology of multiple psychiatric disorders and illustrates the complexity of comorbidity, an area we explore further in this paper.

## 4. Substance Use Disorder

### 4.1. PTSD as a Risk Factor of SUD

Substance use disorders are characterized by repeated misuse of drugs despite negative consequences [76]. Childhood adversity leads to a greater risk for substance misuse and escalation to a substance use disorder (SUD) [77]. Recent studies have found that specific events during development, like childhood abuse, are associated with progression to marijuana use [78,79]. Those who were physically and sexually abused have a 12-fold increase to their risk of regular drinking or marijuana use by the age of 10 [80]. Additionally, childhood maltreatment before the age of 11 raises risk for binge drinking from ages 12–18 years old [81].

An unfortunate, yet common byproduct of adverse childhood events is the diagnosis of depression. Studies have found that depression has a bidirectional relationship with SUDs [82,83,84]. In fact, those with early life stress have less success with SUD treatments and are at a greater risk of relapsing [85,86,87]. A recent study also found that depression mediated the link between early life stress and binge drinking patterns in adolescents [88].

### 4.2. SUD: Effects on the Limbic System and Links to Its Development

The limbic system supports emotional, motivational, stress, and reward-related behaviors. As such, it plays a profound role in drug misuse. SUDs are associated with profound changes to limbic system function. For example, ingestion of opioids and other substances will in the short term provoke large increases in synaptic concentrations of ventral striatal dopamine, whereas in the long term, chronic substance misuse is associated with blunted dopaminergic signaling and the downregulation of dopamine receptors [89]. Different limbic circuits play distinct roles in reward processing. The ventral tegmental-accumbens circuit is linked with drug-associated reward signals and reward prediction [90]. During drug withdrawal, a decrease in the activity of ventral tegmental dopamine neurons has been observed, with a decrease in the release of dopamine in the nucleus accumbens [90]. Hypoactivation in regulatory regions such as the rostral anterior cingulate cortex and ventromedial prefrontal during tasks of emotion regulation is found in individuals with SUD compared to individuals who do not have SUD. Individuals with SUD rarely exhibit hyperactivation in emotion-processing regions when emotionally provoked [91]. In contrast, the hippocampal-extended amygdala circuit has been more often associated with memory of significant stimuli and conditioned responses with drug exposure [90]. These circuits have been implicated in substance misuse in youth; the hippocampus and amygdala volume are smaller in adolescents with SUD in comparison to controls [92].

Another study looked at the impact sex differences may have on alcohol effects. Compared to controls, both males and females showed lower gray matter volume in the frontal and temporal areas, as well as slower white matter volume growth [93]. This study also controlled for comorbid substance use, providing evidence that these findings are primarily attributed to the effects of alcohol. Similarly, another study found that those who began binge drinking before the age of 21 had altered white matter trajectories in frontal regions [94]. Since the temporal lobe is crucial for learning and memory and the frontal lobe for executive functioning and systematic decision-making, it is important to understand how these brain circuits are affected by the development of an SUD [95,96].

### 4.3. Brain Network Connectivity

Like other psychiatric disorders, SUD appears to differentially impact specific regions of the brain. In an fMRI study, college-aged participants without SUD were given a monetary task and a social reward task. Using neuromelanin-sensitive MRI (NM-MRI), it was observed that lower amounts of midbrain dopamine were correlated with substance misuse patterns [97]. Interestingly, those who were given positive social feedback from the social reward task had greater NM-MRI signals, indicating the role of positive sociality during adolescence and the relationship with drug engagement. This finding lends support to the theory that lower dopamine function increases the risk for SUDs. Another longitudinal study used fMRI to scan adolescents before they began using drugs and alcohol, finding that compared to those who had refrained from drinking, those who later became heavy drinkers had less activation in frontal brain regions during a go/no-go inhibition task [98,99]. As these individuals progressed into heavy drinkers, brain activation increased in this group, suggesting they may require more activation to perform a task than others.

## 5. Brain Regions Linked to Both PTSD and SUD

Here we focus on how alterations in memory neural pathways may be a common underpinning of PTSD and SUDs in adolescents. Several brain regions are implicated in both PTSD and SUD, specifically the hippocampus and amygdala.

In youth with PTSD, reduced brain volumes have been observed, and three foundational studies observed reduced intracranial and cerebral volumes in youth with PTSD [100,101,102]. An overwhelming level of stress in youth can result in adverse brain development. For example, PTSD subjects experienced smaller total midsagittal area of the corpus callosum and middle and posterior regions, and larger left, right, and total lateral ventricles than controls [101]. Further, superior temporal gyrus gray matter volumes were larger, and white matter volumes were smaller in subjects with PTSD compared to controls [102]. The hippocampus, which is crucial for memory and new learning [103] had lower volume in adults with a childhood history of abuse compared to those without a history of abuse [104], which might contribute to disrupted memory regulation in PTSD [103].

Early-life stress was associated with impaired cognitive control in adolescence along with hyperactivation of the posterior insula/claustrum in participants given a task that required divided attention [105].

Adolescents may use substances to cope with PTSD and those substances can also impact brain health. Cannabis can be utilized for coping with PTSD to self-medicate for symptoms [106]. For instance, individuals that misuse solvents through inhalation show cerebral and cerebellar hyperintensities with MRI [107]. Further, participants with SUD displayed less working memory task-related activation in the orbitofrontal cortex [108,109].

Langenecker et al.’s randomized controlled trial displays an association between two biomarkers of potentially increased risk for an SUD [110]. If there was an anticipation of a major monetary win, there was activation in both the left and right amygdala, which was positively associated with the intensity of euphoria in response to d-amphetamine administration [110].

In summary, individuals with PTSD and SUD show abnormalities in brain structures along the memory neural pathways, which might ultimately impact the function of these regions. Altered function in these regions may in turn impact behaviors relevant for PTSD and SUD symptomatology, including valuation of rewards and impulse control.

## 6. Epigenetics

### How Stress and Drug Use Lead to Epigenetic Changes

Epigenetics are changes in chromatin structure that do not directly alter the gene sequence, only gene expression, which can occur via DNA methylation and histone modification [111,112,113,114]. Epigenetic mechanisms provide experience-dependent regulation of gene expression in pathways that support memory [115].

There has been increased focus on how these mechanisms play a role in lasting memories of trauma and conditioning to addictive drugs and drug-associated cues [115]. Epigenetic changes are present in SUDs, for example, chronic cocaine administration can increase histone acetylation on H3 and H4 in the nucleus accumbens, a region of the brain involved in reward [116]. Histone deacetylases (HDACs) are a family of enzymes that remove acetyl groups from histones to make chromatin less accessible, repressing gene expression [117]. HDAC5 has activity-dependent regulation in neurons, and its enrichment in the nucleus accumbens is visible [118]. In the transition of drug use to addiction, also seen in chronic stress, there are epigenetic changes in the activity of HDAC5 [117]. PTSD can also result in epigenetic changes, and individuals with early-life trauma have shown methylation, which is a silencing mechanism for the genes encoding glucocorticoid receptors and *bdnf* [119,120].

## 7. Research Gaps

Few studies have focused on the comorbidity of adolescent PTSD and development of an SUD, especially in the past 5 years. Some studies did not investigate PTSD specifically, but rather early life stress. Since PTSD and early-life stress are not interchangeable terms, it becomes challenging to definitively say those results would apply to adolescents diagnosed with PTSD. This review further highlights the need for neuroimaging studies in this comorbid population.

It can be valuable to have studies that specifically address the sex-specific differences of developing an SUD in those diagnosed with PTSD in adolescence. The aim of our review is to investigate the current literature that can establish a connection between the development of an SUD in adolescents who have PTSD. With this connection being further studied, along with the sex-specific differences in the likelihood of developing an SUD, new therapy methods can focus on preventing the occurrence of SUDs in adolescents with PTSD. Additionally, public health interventions can be implemented in society to alleviate the long-lasting symptoms of trauma.

Most of the current literature focuses on alcohol or cannabis use disorders in adolescents. Less research targets adolescents who have misused other substances such as nicotine, opiates, stimulants, or benzodiazepines. With the current NIH-funded longitudinal study, Adolescent Cognitive Brain Development (ABCD), we will soon be able to better understand the long-term neural effects and resulting behaviors of drug use during developmental years. In conclusion, we identified a lack of longitudinal studies using neuroimaging that examined markers for developing SUD in adolescents with PTSD.

## 8. Conclusions

PTSD during adolescence can influence brain development, behavior, and memory. For adolescents diagnosed with PTSD, chronic substance use may be a coping mechanism to alleviate the effects of trauma. In this review, we identified neuroimaging studies that displayed the changes that PTSD, trauma, or SUD can cause in the adolescent brain. We also briefly considered the epigenetic changes that each can cause in adolescents. Despite there being an abundance of literature on PTSD and SUD in adolescents, there was little recent literature that utilized neuroimaging tools to study the comorbidities of PTSD and SUD in adolescents. It raises questions as to how changes in neural pathways of youth diagnosed with PTSD can place them at a higher risk for developing an SUD following the diagnosis, the time between the diagnosis of PTSD and the development of an SUD, sex-specific differences, and the risk factors in adolescents with PTSD that would make them more likely than similarly diagnosed peers to develop an SUD. Future studies can focus on studying the neural changes that occur in adolescents diagnosed with PTSD with and without an SUD. Using neuroimaging to identify the linkage between the development of SUD in individuals diagnosed with PTSD will provide useful. As there are likely a multitude of factors and determinants that play a role in PTSD and SUDs, we hope this review prompts further research to identify potential answers to these questions and provoke the thought of pushing biomedical research towards a more public health approach.

## Data Availability

No new data were created or analyzed in this study. Data sharing is not applicable to this article.
